# Pain in Functional Motor Disorders: Clinical Correlates From the Italian Registry

**DOI:** 10.1111/ene.70634

**Published:** 2026-05-18

**Authors:** Veronica Nisticò, Benedetta Demartini, Christian Geroin, Enrico Marcuzzo, Angela Sandri, Luigi Michele Romito, Roberto Eleopra, Lucia Tesolin, Francesco de Bertoldi, Alessandra Nicoletti, Giovanni Mostile, Nicola Modugno, Enrica Olivola, Andrea Pilotto, Alessandro Magliozzi, Tommaso Ercoli, Giovanni Defazio, Martina Petracca, Carlo Dallocchio, Rosa De Micco, Marcello Esposito, Roberto Erro, Roberto Ceravolo, Michele Tinazzi

**Affiliations:** ^1^ Aldo Ravelli Research Center for Neurotechnology and Experimental Brain Therapeutics University of Milan Milan Italy; ^2^ Department of Health Sciences University of Milan Milan Italy; ^3^ SC Psichiatria ASST Santi Paolo e Carlo, Presidio San Paolo Milan Italy; ^4^ Department of Surgery, Dentistry, Pediatrics, and Gynecology University of Verona Verona Italy; ^5^ Neurology Unit, Movement Disorders Division, Department of Neurosciences, Biomedicine and Movement Sciences University of Verona Verona Italy; ^6^ Fondazione IRCCS Istituto Neurologico Carlo Besta Department of Clinical Neurosciences, Parkinson and Movement Disorders Unit Milan Italy; ^7^ FND Outpatients Clinic, Neurology and Stroke Unit General Hospital of Bolzano Bolzano Italy; ^8^ Department of Psychiatry General Hospital of Bolzano Bolzano Italy; ^9^ Department G.F. Ingrassia, Section of Neurosciences University of Catania Catania Italy; ^10^ Oasi Research Institute – IRCCS Troina Italy; ^11^ IRCCS Neuromed Pozzilli Italy; ^12^ Department of Clinical and Experimental Sciences, Neurology Unit University of Brescia Brescia Italy; ^13^ Laboratory of Digital Neurology and Biosensors University of Brescia Brescia Italy; ^14^ Neurology Unit, Department of Continuity of Care and Frailty ASST Spedali Civili Brescia Hospital Brescia Italy; ^15^ Neurobiorepository and Laboratory of Advanced Biological Markers University of Brescia and ASST Spedali Civili Hospital Brescia Italy; ^16^ Neurological Unit AOU Sassari, University of Sassari Sassari Italy; ^17^ Department of Translational Biomedicine and Neuroscience Aldo Moro University of Bari Bari Italy; ^18^ Fondazione Policlinico Universitario Agostino Gemelli IRCCS Rome Italy; ^19^ SC Neurologia, Dipartimento Di Area Medica Specialistica ASST Pavia Pavia Italy; ^20^ Department of Advanced Medical and Surgical Sciences University of Campania “Luigi Vanvitelli” Naples Italy; ^21^ Clinical Neurophysiology Unit Cardarelli Hospital Naples Italy; ^22^ Department of Medicine, Surgery and Dentistry “Scuola Medica Salernitana” University of Salerno Baronissi (SA) Italy; ^23^ IRCCS Synlab SDN Naples Italy; ^24^ Centro Clinico Malattie NeuroDegenerative Azienda Ospedaliero Universitaria Pisana Pisa Italy

**Keywords:** functional movement disorders, functional neurological disorders, Italian registry, pain, quality of life

## Abstract

**Background:**

Pain is a frequent and disabling symptom in patients with Functional Motor Disorders (FMD), yet its clinical correlates and impact have not been characterized in large, representative cohorts. The aims of this study were to identify clinical predictors and correlates of pain intensity and interference among FMD patients.

**Methods:**

We conducted a cross‐sectional analysis within the Italian Registry of Functional Motor Disorders, including 466 adults with FMD recruited from 25 movement disorders centers. Pain was assessed using the Brief Pain Inventory (BPI), which captures pain experienced during the 24 h prior to assessment, alongside sociodemographic, clinical, and psychometric data on depression, anxiety, alexithymia, fatigue, and quality of life.

**Results:**

Pain was reported by 78.8% of participants. Fibromyalgia strongly predicted pain, whereas comorbid epilepsy was linked to reduced risk. The presence of pain was associated with a poorer self‐reported sense of physical health. Among patients with pain, higher intensity and interference were associated with younger age, Restless Legs Syndrome, Irritable Bowel Syndrome, physical trauma as a precipitating factor for FMD, anxiety, and poorer physical and mental health. Painkillers were prescribed to 41.6% of patients, yet 39.27% reported pain despite medications, and 39.48% had pain while not receiving analgesics.

**Conclusions:**

Pain is highly prevalent in FMD when assessed over the previous 24 h and is frequently reported in patients who are either not receiving analgesics or continue to experience pain despite treatment. Multiple factors influence its intensity and interference in daily life. Longitudinal studies are needed to clarify causal relationships and to inform pain management strategies.

## Graphical abstract

We analyzed pain intensity, interference, and clinical correlates in patients with Functional Motor Disorders (FMD) from the multicenter Italian Registry of FMD. Pain was highly prevalent (78.8%) and associated with fibromyalgia, restless leg syndrome, irritable bowel syndrome, physical trauma as a precipitating factor, younger age, anxiety, and reduced quality of life. Many patients reported pain despite or without analgesic treatment, highlighting gaps in management and the need for longitudinal studies.

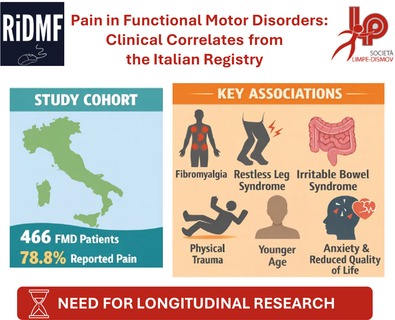

## 1 Introduction

Functional Motor Disorders (FMD) are among the most common manifestations of Functional Neurological Symptom Disorder (FND), characterized by an altered voluntary motor and/or sensory function that cannot be explained by typical neurological diseases or other medical conditions [1, 2]. FMD phenomenology encompasses a broad spectrum of disturbances such as tremor, jerks, tics, dystonia, parkinsonism, weakness, gait, and speech disorders, appearing in isolated form or in a variety of their combinations [3]. Although FMDs are potentially reversible, their pathophysiology remains poorly understood, and they are a significant cause of disability, reduced quality of life, and burden on national health services [3, 4, 5].

Among the associated symptoms, pain is frequently reported by patients with FMD [6]. Pain is defined as an unpleasant sensory and emotional experience associated with actual or potential tissue damage, or resembling such experience [7]. In fact, the debate is ongoing regarding whether pain should be included in the diagnostic criteria for FND, either as a distinct functional sensory symptom (so‐called “nociplastic pain”, which arises from altered nociception despite the absence of tissue damage [8]) or as a diagnostic specifier [9]. A recent systematic review with meta‐analysis estimated that 55% of patients with FND report either pain symptoms or chronic pain, which may be accompanied by other chronic pain comorbidities such as complex regional pain syndrome, irritable bowel syndrome (IBS), and fibromyalgia [6].

Results so far come from mostly small, single‐center cohorts, generally addressing FND as a broad entity. Here, we conducted a cross‐sectional multicenter study in a large Italian cohort of patients with a definite diagnosis of FMD. Our study aimed to: (i) describe pain in FMD and identify its independent clinical predictors by comparing patients with and without pain; (ii) within the subgroup of patients reporting pain, explore the demographic and clinical correlates of pain severity, focusing on specific motor phenotypes, associated non‐motor symptoms, certified neurological, non‐neurological, and psychiatric comorbidities, as well as the impact of pain on quality of life. Our study provides the first large‐scale, cross‐sectional, multicenter evaluation focused exclusively on FMD, adopting a comprehensive approach that integrates pain assessment with the standardized evaluation of other relevant clinical and psychosocial determinants.

## 2 Materials and Methods

Data were obtained from the Italian Registry of Functional Motor Disorders (IRFMD), a multicenter initiative that includes 25 Italian centers, managed by the Department of Neurosciences, Biomedicine and Movement Sciences, University of Verona, by the Italian Academy for the Study of Parkinson's Disease and Other Movement Disorders, and Fondazione LIMPE. Approval was obtained from the Institutional Ethics Committee of the Coordinating Center (University of Verona, AOUI, Prog. 1757CESC) and confirmed by the Committees of each participating center. The data collection was conducted between January 1, 2020, and December 31, 2022. The IRFMD full methodology has been described elsewhere [10].

The IRFMD included patients with a diagnosis of clinically definite FMDs based on Gupta and Lang's criteria [11]. Demographic and clinical variables were obtained by a structured interview that included age, gender, FMD disease duration, neurological phenotype (i.e., tremor, weakness, etc.), FMD onset, additional functional symptoms (i.e., functional seizures/spells), presence of diagnosed psychiatric, neurological, and other medical comorbidities [10, 12].

One section of the IRFMD investigated the experience of pain using the Brief Pain Inventory [13]. The BPI measures pain severity—four items scored from 0 (no pain) to 10 (worst pain)—and pain interference—seven items scored from 0 (no interference) to 10 (complete interference), reflecting the extent to which pain disrupts daily functioning, including general activity, mood, walking ability, work, relations with other people, sleep, and enjoyment of life—occurring in the 24‐h prior to the assessment. Scores are calculated as the sum of the respective items for each section [14].

Moreover, the same IRFMD section includes: The Simplified Functional Movement Disorders Rating Scale (S‐FMDRS) investigating the severity of FMD; the Beck Depression Inventory‐II (BDI‐II) [15] and the Beck Anxiety Inventory (BAI [16]), to respectively assess the levels of depressive and anxiety symptoms; the Toronto Alexithymia Scale 20‐items version (TAS‐20), investigating patients' difficulties in identifying and describing their own feelings [17]; the Short‐form‐12 Health Survey Questionnaire, measuring the patients' perceived physical and mental health (SF‐12 [18]); the Multidimensional Fatigue Inventory (MFI‐20), which measures fatigue across five domains: General fatigue, physical fatigue, reduced activity, reduced motivation, and mental fatigue [19]. For the purposes of this study, exclusion criteria were: (1) age < 20 years old [20], as this range includes both children (< 10 years) and adolescents (10–19 years), in whom pain may be expressed differently compared to adults [21]; (2) incomplete BPI data.

### 2.1 Statistical Analysis

Statistical analyses were performed in SPSS‐29. Data are expressed as mean ± standard deviation for continuous variables and counts and percentages for categorical variables. The significant threshold for inferential analysis was set at α ≤ 0.05; all tests were two‐tailed. Non‐parametric tests were implemented, as the Kolmogorov–Smirnov test revealed a non‐normal distribution of the variables.

First, descriptive statistics for sociodemographic, psychometric, and clinical variables were analyzed.

Second, we classified the sample into patients with pain vs. without pain based on the BPI score and compared the two groups across all other variables using the Mann–Whitney U test for continuous variables and chi‐squared test for categorical variables. Subsequently, to identify predictors of pain presence, we conducted a logistic regression analysis. Candidate independent variables were selected based on significant univariate group differences, together with conceptual and clinical relevance, while avoiding redundancy among closely related predictors. In particular, the model included variables reflecting FMD phenomenology, comorbidities, trauma history, and health status that were considered most clinically informative and statistically appropriate. Analgesic use and general medication variables, although significant at the univariate level, were not included in the multivariable model due to their heterogeneity and the lack of temporal information regarding prescription (i.e., whether medication preceded or followed pain onset), which limits causal interpretability. These variables are therefore discussed separately in the manuscript. Adjusted odds ratios with 95% CI for the presence of pain were estimated.

Third, in the subset of patients reporting pain, the BPI Intensity and Interference subscales were treated as continuous variables. Exploratory univariate analyses (Mann–Whitney U test for group comparisons and Spearman's correlations for continuous variables) were performed to examine associations between pain ratings and sociodemographic, psychometric, and clinical variables (Appendix S1 for results). Variables showing significant univariate associations and deemed conceptually and clinically meaningful were entered in multivariate regression models to identify independent predictors of pain intensity and interference. These linear models were designed to identify predictors of pain severity among patients who already reported pain and, therefore, address a question distinct from the logistic model on pain presence. Effect magnitude was categorized according to standardized beta coefficients [22, 23]: |β| < 0.10 = Very Small; 0.10 ≤ |β| < 0.20 = Small; 0.20 ≤ |β| < 0.30 = Small‐to‐Moderate; 0.30 ≤ |β| < 0.40 = Moderate; 0.40 ≤ |β| < 0.50 = Moderate‐to‐Large; |β| ≥ 0.50 = Large. Clinical relevance was interpreted using the Minimally Clinically Important Difference (MCID) thresholds [24, 25], defined as the smallest change in a symptom score perceived as meaningful by the patient. For pain intensity, we referred to prior literature indicating that clinically meaningful change generally corresponds to approximately 10%–15% of the scale range; accordingly, on the 0–40 BPI intensity scale, this corresponds to about a 4‐point change in the B coefficient for Pain Intensity. For pain interference, no directly established MCID was available for our specific analytic context; therefore, we adopted an approximate threshold based on 10% of the Pain Interference scale range (0–70), corresponding to about a 7‐point change in the B coefficient for Pain Interference. To account for sampling variability, effects were considered clinically meaningful when either the point estimate or, more conservatively, the lower bound of the interval defined by B ± Standard Error exceeded the MCID.

## 3 Results

### 3.1 Sociodemographic and Clinical Information

From a total of 500 patients included in the IRFMD, 466 eligible participants were retained after applying exclusion criteria. Of these, 347 (74.5%) were female. Mean age was 47.2 ± 15.2 (range: 20–78), and mean years of education was 12.21 ± 3.64. Mean disease duration was 4.9 years, and 114 (24.5%) patients had stopped working due to FMD, on average for 2.9 months. Most patients (*N* = 290, 62.2%) presented with two or more combined functional motor symptoms, whereas 176 (37.8%) presented with a single functional motor symptom. Based on the BPI, 367 patients (78.8%) reported experiencing pain, while 99 (21.2%) did not. In the overall sample, the average Pain Intensity score was 14.67 ± 11.51 (out of a maximum of 40), and the average Pain Interference score was 24.15 ± 20.62 (out of a maximum of 70). Further sociodemographic, clinical, and psychometric information is reported in Table 1.

**TABLE 1:** Demographic and clinical features of FMD patients with and without pain.

	Overall sample *n* = 466	No pain *n* = 99 (21.2%)	Pain *n* = 367 (78.8%)	*p*
**Demographics and clinical characteristics**				
Age, mean (SD)	47.2 (15.2)	45.7 (17.7)	47.6 (14.5)	0.222
Sex, (M/F; *n*, %)	119 (25.5)/347 (74.5)	23 (23.2)/76 (76.8)	96 (26.2)/271 (73.8)	0.554
Education (years), mean (SD)	12.2 (3.6)	12.5 (3.5)	12.1 (3.7)	0.278
Interruption of Work Activities, *N* (%)	114 (24.5)	17 (17.2)	97 (26.4)	0.057
Disease duration (years), mean (SD)	4.9 (7.7)	4.8 (9.2)	4.9 (7.2)	**0.037**
S‐FMDRS, mean (SD)	14 (9.3)	10.41 (7.28)	14.92 (9.4)	**< 0.001**
BPI – Pain intensity, mean (SD)	14.7 (11.5)	0	14.67 (11.5)	NA
BPI – Pain interference, mean (SD)	24.2 (20.6)	0	24.15 (20.6)	NA
BPI – Total score, mean (SD)	38.8 (30.6)	0	38.82 (30.6)	NA
Childhood trauma–only physical, *N*, (%)	39 (8.4)	5 (5.05)	34 (9.3)	0.179
Childhood trauma–only psychological, *N* (%)	65 (14)	8 (8.1)	57 (15.5)	0.058
Childhood trauma–combined physical & psychological, *N* (%)	23 (5)	1 (1)	22 (6)	**0.042**
Family history of neurological Disease, *N* (%)	131 (28.1)	25 (25.3)	106 (29)	0.476
Friends with neurological Disease, *N* (%)	34 (7.3)	8 (8.1)	26 (7.1)	0.735
Family history of psychiatric Disease, *N* (%)	39 (8.4)	9 (9.1)	30 (8.2)	0.770
**Functional movement disorder phenotype**, *N* (%)				
FMD, isolated/combined presentation	Isolated: 176 (37.8) Combined: 290 (62.2)	Isolated: 51 (51.5) Combined: 48 (48.5)	Isolated: 125 (34.1) Combined: 242 (65.9)	**0.001**
Tremor	186 (39.9)	38 (38.4)	148 (40.3)	0.726
Facial movement disorder	74 (15.9)	12 (12.1)	62 (16.9)	0.249
Tics	20 (4.3)	5 (5.1)	15 (4.1)	0.675
Jerks	62 (13.3)	7 (7.1)	55 (15)	**0.040**
Dystonia	110 (23.6)	12 (12.1)	98 (26.7)	**0.002**
Weakness	259 (55.6)	47 (47.5)	212 (57.8)	0.067
Parkinsonism	19 (4.1)	3 (3)	16 (4.4)	0.553
Gait disorder	196 (42.1)	36 (36.4)	160 (43.6)	0.196
Speech disorder	90 (19.3)	19 (19.2)	71 (19.4)	0.972
Swallowing disorder	43 (9.2)	7 (7.1)	36 (9.8)	0.403
**Associated functional neurological symptoms**, *N* (%)				
Any functional neurological symptom	280 (60.1)	45 (45.5)	235 (64)	**0.001**
Functional seizures	62 (13.3)	10 (10.1)	52 (14.2)	0.290
Visual functional symptoms	76 (16.3)	9 (9.1)	67 (18.3)	**0.028**
Cognitive functional symptoms	95 (20.4)	14 (14.1)	81 (22.1)	0.082
Sensory functional symptoms	157 (33.7)	26 (26.3)	131 (35.7)	0.078
Fibromyalgia	71 (15.2)	3 (3)	68 (18.5)	**< 0.001**
Irritable bowel syndrome	21 (4.5)	5 (5.1)	16 (4.4)	0.769
**Psychiatric comorbidities (clinical diagnoses)**, *N* (%)				
Any psychiatric diagnosis	179 (38.4)	28 (28.3)	151 (41.2)	**0.020**
Schizophrenia	9 (1.9)	0 (0)	9 (2.5)	0.116
Bipolar disorder	13 (2.8)	1 (1)	12 (3.3)	0.226
Major Depression	74 (15.9)	11 (11.1)	63 (17.2)	0.144
Anxiety disorder	99 (21.3)	17 (17.2)	82 (22.3)	0.264
Impulse control disorder/OCD	16 (3.4)	3 (3)	13 (3.5)	0.804
Post‐traumatic stress disorder	3 (0.6)	0 (0)	3 (0.8)	0.367
Dissociative fugue state	6 (1.3)	2 (2)	4 (1.1)	0.466
Somatization disorder	23 (4.9)	4 (4)	19 (5.2)	0.643
Eating disorder	12 (2.6)	3 (3)	9 (2.5)	0.747
Sexual disorder	5 (1.1)	0 (0)	5 (1.4)	0.243
Personality disorder	19 (4.1)	2 (2)	17 (4.6)	0.244
**Neurological comorbidities (clinical diagnoses)**, *N* (%)				
Any neurological comorbidity	151 (32.4)	24 (24.2)	127 (34.6)	0.051
Multiple sclerosis	7 (1.5)	0 (0)	7 (1.9)	0.166
Parkinson's Disease/Parkinsonism	6 (1.3)	0 (0)	6 (1.6)	0.200
Hyperkinetic movement disorders	13 (2.8)	1 (1)	12 (3.3)	0.226
Neuropathy	18 (3.9)	2 (2)	16 (4.4)	0.284
Epilepsy	14 (3)	6 (6.1)	8 (2.2)	**0.045**
Cerebrovascular Disease	31 (6.7)	8 (8.1)	23 (6.3)	0.520
Migraine (clinical diagnosis)	56 (12)	7 (7.1)	49 (13.4)	0.088
Restless legs syndrome	8 (1.7)	0 (0)	8 (2.2)	0.138
**Non‐neurological comorbidities**, *N* (%)				
Any non‐neurological comorbidity	212 (45.5)	34 (34.3)	178 (48.5)	**0.012**
Hypertension	81 (17.4)	16 (16.2)	65 (17.7)	0.718
Diabetes mellitus	24 (5.2)	4 (4)	20 (5.5)	0.573
Heart Disease	33 (7.1)	8 (8.1)	25 (6.8)	0.662
Dyslipidemia	50 (10.7)	12 (12.1)	38 (10.4)	0.614
Thyroid Disease	60 (12.9)	12 (12.1)	48 (13.1)	0.801
Arthritis/Rheumatic Disease	29 (6.2)	1 (1)	28 (7.6)	**0.016**
Tumor	20 (4.3)	5 (5.1)	15 (4.1)	0.675
Gastroenterological disorder	20 (4.3)	3 (3)	17 (4.6)	0.485
**Precipitating factors**, *N* (%)				
Any precipitating factor	261 (56)	54 (54.6)	207 (56.4)	0.741
Physical trauma	90 (19.3)	15 (15.2)	75 (20.4)	0.237
Psychological trauma	116 (25)	22 (22.2)	94 (25.6)	0.489
Surgery	68 (14.6)	10 (10.1)	58 (15.8)	0.154
General anesthesia	30 (6.4)	4 (4)	26 (7.1)	0.273
Adverse drug reaction	36 (7.7)	8 (8.1)	28 (7.6)	0.881
Infection	15 (3.2)	3 (3)	12 (3.3)	0.905
Panic attack	17 (3.7)	6 (6.1)	11 (3)	0.149
Fugue state	15 (3.2)	2 (2)	13 (3.5)	0.446
**Therapies received**, *N* (%)				
Physical rehabilitation	194 (41.6)	34 (34.3)	160 (43.6)	0.097
Cognitive‐behavioral therapy	80 (17.2)	12 (12.1)	68 (18.5)	0.134
Hypnosis	10 (2.2)	2 (2)	8 (2.2)	0.923
Transcranial magnetic stimulation	11 (2.4)	1 (1)	10 (2.7)	0.319
Botulinum toxin	40 (8.6)	3 (3)	37 (10.1)	**0.026**
Speech therapy	11 (2.4)	2 (2)	9 (2.5)	0.802
Other therapies	28 (6)	4 (4)	24 (6.5)	0.353
**Medications**, *N* (%)				
Any medication	227 (48.7)	30 (30.3)	197 (53.7)	**< 0.001**
Antipsychotics	31 (6.7)	4 (4)	27 (7.4)	0.240
Benzodiazepines	106 (22.8)	13 (13.1)	93 (25.3)	**0.010**
Antidepressants	133 (28.5)	14 (14.1)	119 (32.4)	**< 0.001**
Antiepileptic drugs	86 (18.5)	9 (9.1)	77 (21)	**0.007**
**Painkillers**, *N* (%)				
Any painkiller	194 (41.6)	11 (11.1)	183 (49.9)	**< 0.001**
NSAIDs	118 (25.3)	5 (5.1)	113 (30.8)	**< 0.001**
Steroids	16 (3.4)	1 (1)	15 (4.1)	0.136
Opioids	37 (7.9)	1 (1)	36 (9.8)	**0.004**
Local anesthetics	2 (0.4)	0 (0)	2 (0.5)	0.462
SSRIs	45 (9.7)	1 (1)	44 (12)	**0.001**
Anti‐epileptic medication	46 (9.9)	0 (0)	46 (12.5)	**< 0.001**
**Psychometric scales, mean (SD)**				
BDI‐II	13.9 (10.7)	10.2 (9.5)	15 (10.8)	**< 0.001**
BAI	20.3 (11.5)	14.3 (11.6)	21.9 (11)	**< 0.001**
TAS‐20 Total Score	53.5 (14.6)	48.1 (14)	54.9 (14.5)	**< 0.001**
MFI‐20 Total Score	62.8 (4.3)	54.1 (19.9)	65.1 (16.1)	**< 0.001**
SF‐12 Physical health	34.1 (11.3)	42.7 (10.8)	31.9 (10.4)	**< 0.001**
SF‐12 Mental health	41.9 (12)	44.5 (12.1)	41.2 (12)	**0.01**

*Note:* Missing data: BAI: 2; TAS‐20: 16; SF‐12: 1; MFI‐20: 27.

Abbreviations: BAI, Beck Anxiety Inventory; BDI‐II, Beck Depression Inventory – second version; F, Female; M, Male; MFI‐20, Multidimensional Fatigue Inventory – 20 Items; *N* (%), Numerosity (percentage); OCD, Obsessive‐Compulsive Disorder; S‐FMDRS, Short Version – Functional Motor Disorder Rating Scale; SF‐12, Short‐Form 12 Item; SD, Standard deviation; TAS‐20, Toronto Alexithymia Scale – 20 Items. At the psychometric scales, higher score means, respectively: higher depressions levels; higher anxiety levels; higher alexithymia levels; higher fatigue; better physical and mental health. *p*‐values refer to comparisons between patients with and without pain; bold indicates significant values (*p* ≤ 0.05).

### 3.2 Factors Associated With the Presence of Pain

Comparing sociodemographic and clinical variables between patients with and without pain, no significant differences emerged in age, biological sex, education, and employment status (all *p* > 0.05, Table 1). Patients with pain were significantly more likely to exhibit combined FMD symptoms rather than isolated ones (χ^2^(1) = 10.107, *p* = 0.001); they showed significantly greater FMD symptom severity, indicated by higher S‐FMDRS total scores (*p* < 0.001), and reported a longer symptom duration (*p* = 0.037). Jerks (χ^2^(1) = 4.235, *p* = 0.040) and dystonia (χ^2^(1) = 9.193, *p* = 0.002) were significantly more frequent in patients with pain than in patients without. Participants with pain also more frequently reported other associated functional symptoms (χ^2^(1) = 11.221, *p* = 0.001), particularly visual functional symptoms (χ^2^(1) = 4.798, *p* = 0.028) and fibromyalgia (χ^2^(1) = 14.501, *p* < 0.001). FMD patients with pain were more likely to have a psychiatric diagnosis (χ^2^(1) = 5.452, *p* = 0.020) and to report both psychological and physical trauma (χ^2^(1) = 4.128, *p* = 0.042) in their history; they were less likely to have an epilepsy diagnosis (χ^2^(1) = 4.03, *p* = 0.045), and they showed a higher prevalence of non‐neurological comorbidities (χ^2^(1) = 6.303, *p* = 0.012), particularly arthritis and rheumatic diseases (χ^2^(1) = 5.854, *p* = 0.016).

Significant differences were also observed in treatment patterns: Patients with pain more frequently received botulinum toxin (χ^2^(1) = 4.941, *p* = 0.026) and pharmacological treatments (χ^2^(1) = 17.052, *p* < 0.001) including benzodiazepines (χ^2^(1) = 6.614, *p* = 0.010), antidepressants (χ^2^(1) = 12.780, *p* < 0.001), and antiepileptics (χ^2^(1) = 7.324, *p* = 0.007).

Six categories of medications used for pain management were identified, prescribed alone or in combination: Non‐steroidal anti‐inflammatory drugs (NSAIDs), steroids, opioids, local anesthetics, selective serotonin reuptake inhibitors (SSRIs), and antiepileptic medications. The use of analgesic medications was significantly more frequent among FMD patients with pain (χ^2^(1) = 48.186, *p* < 0.001), especially NSAIDs (χ^2^(1) = 27.317, *p* < 0.001), opioids (χ^2^(1) = 8.259, *p* = 0.004), SSRIs (χ^2^(1) = 10.773, *p* = 0.001), antiepileptic drugs (χ^2^(1) = 13.768, *p* < 0.001). Overall, 194 (41.6%) patients received at least one drug for pain management. Among the 466 participants, 88 (18.88%) reported no pain and were not taking any analgesic medication, while 11 (2.36%) individuals reported no pain while taking analgesic medication. In contrast, 184 (39.5%) participants reported experiencing pain but were not on any painkillers, and 183 (39.3%) participants reported pain despite taking painkillers. These patterns should be interpreted cautiously, as the absence of analgesic treatment does not necessarily imply undertreatment, but may also reflect contraindications, patient preference, or the use of non‐pharmacological management strategies (e.g., physiotherapy).

Of note, among participants with epilepsy (*N* = 14), 8 patients were not taking antiepileptic drugs, of whom 5 reported pain, whereas 6 patients were receiving antiepileptic treatment, of whom 3 reported pain. No significant association was observed between antiepileptic drug use and pain presence (χ^2^(1) = 0.219, *p* = 0.64) in patients with epilepsy.

Finally, patients reporting pain scored significantly higher on measures of depression (BDI‐II), anxiety (BAI), alexithymia (TAS‐20), and fatigue (MFI‐20), and reported poorer physical and mental health (SF‐12), compared to those without pain (all *p* < 0.01; see Table 1).

In the logistic regression model (χ^2^(15) = 77.554, *p* < 0.001; Nagelkerke R^2^ = 0.28), overall classification accuracy was 83%. Comorbid fibromyalgia was a significant predictor of pain (B = 1.620, *p* = 0.035), with affected patients being more than five times as likely to report pain (OR = 5.05, 95% CI [1.12, 22.83]). Conversely, comorbid epilepsy showed a significant negative association (B = –1.659, *p* = 0.013), with epileptic patients being about 81% less likely to report pain (OR = 0.19, 95% CI [0.05, 0.71]). Higher alexithymia scores (TAS‐20) were also independently associated with pain presence (B = 0.024, *p* = 0.047; OR = 1.02, 95% CI [1.00, 1.05]). Finally, lower SF‐12 Physical Health scores (i.e., poorer perceived physical health) predicted pain presence (B = –0.058, *p* < 0.001; OR = 0.94, 95% CI [0.92, 0.97]). No other predictors reached statistical significance (all *p* > 0.05) (Table 2).

**TABLE 2:** Independent factors associated with the presence of pain in patients with FMD.

	B (SE)	Wald χ	df	*p*	Exp(B)	95% C.I. [LB; UB]
**Disease duration (years)**	−0.034 (0.019)	3.247	1	0.072	0.967	[0.931; 1.003]
**S‐FMDRS**	0.022 (0.020)	1.246	1	0.264	1.022	[0.984; 1.062]
**FMD, single vs. combined** ^**a**^ **presentation**	0.234 (0.306)	0.586	1	0.444	1.264	[0.694; 2.300]
**Visual functional symptoms, yes vs. no** ^**a**^	0.299 (0.470)	0.405	1	0.524	1.349	[0.537; 3.391]
**Fibromyalgia, yes vs. no** ^**a**^	1.620 (0.769)	4.432	1	**0.035**	5.053	[1.118; 22.832]
**Any psychiatric diagnosis, yes vs. no** ^**a**^	0.297 (0.314)	0.894	1	0.344	1.346	[0.727; 2.490]
**Epilepsy, yes vs. no** ^**a**^	−1.659 (0.669)	6.149	1	**0.013**	0.190	[0.051; 0.706]
**Arthritis/Rheumatic Disease, yes vs. no** ^**a**^	1.072 (1.081)	0.984	1	0.321	2.923	[0.351; 24.338]
**Childhood trauma (physical + psychological), yes vs. no** ^**a**^	1.354 (1.159)	1.366	1	0.243	3.872	[0.400; 37.506]
**BDI‐II**	0.009 (0.023)	0.160	1	0.690	1.009	[0.964; 1.056]
**BAI**	0.019 (0.020)	0.923	1	0.337	1.019	[0.980; 1.060]
**TAS‐20 Total Score**	0.024 (0.012)	3.934	1	**0.047**	1.024	[1.000; 1.048]
**MFI‐20 Total Score**	−0.006 (0.011)	0.308	1	0.579	0.994	[0.973; 1.015]
**SF‐12 Physical health**	−0.058 (0.015)	14.311	1	**< 0.001**	0.943	[0.915; 0.972]
**SF‐12 Mental health**	0.013 (0.017)	0.632	1	0.427	1.013	[0.981; 1.047]

Abbreviations: BAI, Beck Anxiety Inventory; BDI‐II, Beck Depression Inventory – second version; C.I., Confidence Interval (95%); DDF, Difficult Describing Feelings; DIF, Difficult Identifying Feelings; EOT, Externally‐Oriented Thinking; LB, Lower Bound; S‐FMDRS, Simplified Functional Movement Disorder Rating Scale; S.E., Standard error; TAS‐20, Toronto Alexithymia Scale—20 Item; UB, Upper Bound. ; Bold indicates significant values (*p* ≤ 0.05); a : Reference category. Missing: 61 subjects out of 466, reflecting incomplete data collection or reporting across participating centers; effective N used = 405. Overall model (χ²(14) = 74.558, *p* < 0.001; Nagelkerke R² = 0.27).

### 3.3 Factors Predicting Higher Pain Intensity and Interference (Pain Ratings)

Among the subset of patients who reported pain on the BPI (*N* = 367, 78.76%), the average Pain Intensity Score was 18.63 ± 9.8 (range: [0–40]), and the average Pain Interference score was 30.66 ± 18.46 (range: [0–70]).

Distinct from the predictors of pain presence reported above, the following analyses examined which factors predicted the severity and daily‐life impact of pain among patients who already reported pain.

Higher pain intensity was predicted by younger age (B = –0.087, β = −0.130, *p* = 0.009); results indicate that for each additional year of age, pain intensity decreased on average by 0.087 points on the 0–40 scale. This is considerably below the 4‐point MCID and therefore not clinically meaningful. The presence of restless legs syndrome (RLS) (B = 6.889, β = 0.093, *p* = 0.043) predicted an average increase in pain intensity of nearly seven points. The B±1 SE interval [3.50; 10.28] approaches and largely overlaps the MCID threshold of 4 points, suggesting that the estimated effect is potentially clinically meaningful, though the lower bound falls just below the threshold. Physical trauma as a precipitating factor (B = 2.949, β = 0.119, *p* = 0.009) also predicted higher pain, with an average increase of about three points (close to the MCID threshold). Higher anxiety levels (BAI; B = 0.164, β = 0.181, *p* = 0.005) predicted higher pain intensity, but the effect per 1‐point BAI increase is 0.164, also below the MCID. Conversely, better physical health (SF‐12 Physical; β = −0.398, B = –0.379, *p* < 0.001) predicted lower pain intensity, indicating a decrease of approximately 0.38 points for each one‐point increase in the SF‐12 Physical score (also below the MCID). The overall model was significant, F(14, 333) = 12.75, *p* < 0.001, and explained 33.1% of the variance in pain intensity (adjusted R^2^ = 0.331) (Table 3).

**TABLE 3:** Independent factors associated with BPI pain intensity.

	B (SE)	β	95% C.I. [LB; UB]	t	*p*	Effect size, based on β	MCID, based on b ± SE
Age	**−0.087 (0.033)**	**−0.130**	[−0.152; −0.022]	−2.644	**0.009**	Small	Below MCID
Work activity interrupted	1.458 (1.096)	0.065	[−0.698; 3.615]	1.330	0.184	NA	NA
S‐FMDRS	0.095 (0.053)	0.092	[−0.009; 0.199]	1.796	0.074	NA	NA
Weakness	0.761 (1.037)	0.038	[−1.279; 2.800]	0.734	0.464	NA	NA
Gait disorder	−0.069 (0.950)	−0.003	[−1.937; 1.799]	−0.072	0.942	NA	NA
Childhood trauma‐physical + psychological	0.230 (1.191)	0.006	[−3.532; 3.993]	0.120	0.904	NA	NA
Sensory functional symptoms	0.477 (1.023)	0.023	[−1.535; 2.489]	0.467	0.641	NA	NA
Fibromyalgia	−0.351 (1.168)	−0.014	[−2.648; 1.946]	−0.301	0.764	NA	NA
Restless legs syndrome	6.889 (3.390)	**0.093**	[0.219; 13.559]	2.032	**0.043**	Very Small	Potentially clinically meaningful
Precipitating factors ‐ Physical trauma	2.949 (1.123)	**0.119**	[0.739; 5.159]	2.625	**0.009**	Small	Below MCID
BDI‐II	0.064 (0.059)	0.069	[−0.053; 0.180]	1.077	0.282	NA	NA
BAI	0.164 (0.058)	**0.181**	[0.049; 0.278]	2.803	**0.005**	Small	Below MCID
MFI‐20 Total Score	−0.013 (0.035)	−0.021	[−0.081; 0.055]	−0.367	0.714	NA	NA
SF‐12 Physical health	−0.379 (0.053)	**−0.398**	[−0.482; −0.276]	−7.216	**< 0.001**	Moderate	Below MCID

Abbreviations: B (SE) = unstandardized regression coefficient with corresponding Standard Error. β = standardized regression coefficient. BAI = Beck Anxiety Inventory. BDI‐II = Beck Depression Inventory – second version. C.I. = Confidence Interval (95%). [LB; UB] = [Lower Bound; Upper Bound]. MCID, based on B±SE = Minimally Clinically Important Difference, and it refers to whether the estimated B exceeds the MCID of 4 points; MCID classification was based on the B ± 1 SE interval. TAS‐20 = Toronto Alexithymia Scale – 20 Item. S‐FMDRS = Simplified Functional Movement Disorder Rating Scale. Bold indicates significant values (*p* ≤ 0.05). Missing: 33 subjects out of 367, reflecting incomplete data collection or reporting across participating centers; effective N used = 334.

Greater pain interference was predicted by comorbid IBS (B = 8.314, β = 0.097, *p* = 0.036), corresponding to an average increase of 8.3 points on the 0–70 Pain Interference scale. Although this estimate exceeds the MCID of 7 points, the relatively large standard error (SE = 3.940) results in a wide interval [4.374; 12.254] that includes values below the MCID; therefore, we cannot state that the true effect of IBS is clinically meaningful. Physical trauma as a precipitating factor (B = 5.495, β = 0.118, *p* = 0.010) predicted a 5.5‐point increase in pain interference; this value is below the MCID threshold and therefore not clinically important. Poorer physical and mental health were also associated with higher pain interference. Specifically, higher SF‐12 Physical scores predicted lower pain interference (β = −0.378, B = –0.671, *p* < 0.001), with each 1‐point increase in SF‐12 Physical corresponding to a 0.671‐point reduction (below the MCID). Similarly, higher SF‐12 Mental scores predicted lower pain interference (β = −0.178, B = –0.276, *p* = 0.003), with a 1‐point increase leading to a 0.276‐point reduction in pain interference (also below the MCID). Finally, the severity of anxiety symptoms showed a trend‐level association (BAI; β = 0.124, *p* = 0.067). The model was statistically significant, F(17, 327) = 11.556, *p* < 0.001, and explained approximately 35.4% of the variance (adjusted R^2^ = 0.354) (Table 4). Collinearity diagnostics indicated that tolerance values for all predictors were above 0.4 and variance inflation factor (VIF) values were below 2.4, suggesting that multicollinearity among independent variables was not a concern.

**TABLE 4:** Clinical variables associated with BPI pain interference.

	B (SE)	β	95% C.I. [LB; UB]	t	*p*	Effect size, based on β	MCID, based on b ± SE
Work activity interrupted	3.062 (2.007)	0.073	[−0.887; 7.011]	1.525	0.128	NA	NA
S‐FMDRS	0.062 (0.100)	0.032	[−0.134; 0.258]	0.620	0.535	NA	NA
Childhood trauma‐psychological	1.068 (2.814)	0.022	[−4.469; 6.604]	0.379	0.705	NA	NA
Childhood trauma‐physical + psychological	2.866 (4.196)	0.038	[−5.391; 11.122]	0.683	0.495	NA	NA
Functional Seizures	−1.575 (2.624)	−0.029	[−6.739; 3.589]	−0.600	0.549	NA	NA
Cognitive functional symptoms	0.217 (2.177)	0.005	[−4.066; 4.501]	0.100	0.921	NA	NA
Sensory functional symptoms	0.878 (1.879)	0.023	[−2.820; 4.576]	0.467	0.641	NA	NA
Irritable bowel syndrome	8.314 (3.940)	**0.097**	[0.562; 16.066]	2.110	**0.036**	Very Small	Not clearly clinically important
Migraine	−2.502 (2.494)	−0.046	[−7.408; 2.404]	−1.003	0.316	NA	NA
Any Non‐neurological comorbidity	−1.737 (1.726)	−0.047	[−5.133; 1.660]	−1.006	0.315	NA	NA
Precipitating factors ‐ Physical trauma	5.495 (2.131)	**0.118**	[1.301; 9.689]	2.578	**0.010**	Small	Below MCID
BDI‐II	0.154 (0.116)	0.089	[−0.075; 0.383]	1.326	0.186	NA	NA
BAI	0.210 (0.114)	0.124	[−0.015; 0.435]	1.837	0.067	NA	NA
TAS‐20 Total Score	0.043 (0.068)	0.034	[−0.090; 0.177]	0.638	0.524	NA	NA
MFI‐20 Total Score	0.032 (0.068)	0.029	[−0.102; 0.165]	0.468	0.640	NA	NA
SF‐12 Physical health	−0.671 (0.097)	**−0.378**	[−0.862; −0.480]	−6.910	**< 0.001**	Moderate	Below MCID
SF‐12 Mental health	−0.276 (0.093)	**−0.178**	[−0.459; −0.093]	−2.963	**0.003**	Small	Below MCID

Abbreviations: B (SE) = unstandardized regression coefficient with corresponding Standard Error. BAI = Beck Anxiety Inventory. BDI‐II = Beck Depression Inventory – second version. C.I. = Confidence Interval (95%). [LB; UB] = [Lower Bound; Upper Bound]. MCID, based on B±SE = Minimally Clinically Important Difference, and it refers to whether the estimated B exceeds the MCID of 7 points; MCID classification was based on the B ± 1 SE interval. TAS‐20 = Toronto Alexithymia Scale – 20 Item. S‐FMDRS = Simplified Functional Movement Disorder Rating Scale. Bold indicates significant values (*p* ≤ 0.05). Missing: 39 subjects out of 367, reflecting incomplete data collection or reporting across participating centers; effective N used = 328.

## 4 Discussion

The aim of this study was to explore the prevalence of pain and its sociodemographic and clinical predictors in a large sample of patients diagnosed with FMD, registered in the multicenter IRFMD [10].

In our cohort, pain was reported by 78.8% of participants, as assessed by the BPI. This prevalence is substantially higher than the 61% [95% CI: 49%–72%] estimate reported in the recent meta‐analysis by Steinruecke et al. [6], even exceeding the upper limit of its confidence interval. This discrepancy may reflect methodological differences: Whereas the meta‐analysis drew on small, retrospective studies reliant on health records, our registry employed cross‐sectional, standardized assessments, allowing for a more consistent and potentially more accurate characterization of pain in FMD. Importantly, however, our study captured pain experienced during the 24 h prior to assessment rather than chronic pain specifically, and this difference in timeframe may also have contributed to the higher prevalence observed. Accordingly, comparisons with chronic pain studies should be interpreted with caution.

### 4.1 Clinical Correlates of Pain Presence in Patients With FMD

We found no significant association between sociodemographic characteristics and the presence of pain in patients with FMD. This result is in contrast with findings reported in the meta‐analysis by Steinruecke and colleagues [6], in which pain was more frequently reported by female patients. However, those findings emerged in patients with functional seizures [26] and in a study of functional disorders among civilians following the First World War [27], when the diagnosis of FND did not follow today's criteria.

Multivariate analysis showed that pain was significantly associated with fibromyalgia, with 18.5% of our sample reporting both conditions. This prevalence is consistent with, and slightly exceeds, the upper bound of the meta‐analytic estimate for fibromyalgia and pain in FND populations (10%, 95% CI: 8%–13%) [6], suggesting a potentially stronger link in individuals with FMD. On the other hand, we did not find a significant association with IBS for pain presence, which showed a more variable prevalence in FMD (from 3% to 36%) in previous studies [28, 29].

No comorbid neurological condition independently predicted pain presence (including migraine, which was not reviewed in the meta‐analysis by Steinruecke and colleagues [6]). Interestingly, however, comorbid epilepsy was negatively associated with pain, indicating that patients with both FMD and epilepsy were significantly less likely to report pain. So far, pain and seizures have been thoroughly investigated in previous cohort studies conducted in epilepsy monitoring units, which have consistently shown that chronic pain is more common in patients with functional seizures than in those with epilepsy. For instance, one study reported chronic pain in 9% of patients with functional seizures compared to only 3% in those with epilepsy [30]. In fact, chronic pain has been identified as a key discriminating factor in the differential diagnosis between functional seizures and epilepsy, contributing strongly to a validated diagnostic scoring tool [30]. In this context, our finding that comorbid epilepsy was associated with a lower likelihood of pain in FMD further supports the notion that the presence of epilepsy may represent a clinically distinct phenotype within the broader FND spectrum, with implications for diagnostic and therapeutic strategies. At the same time, this finding should be interpreted cautiously. We cannot exclude that differences in treatment may have contributed to this association, as several antiepileptic drugs are also used for pain modulation. In our sample, the absence of a significant association between antiepileptic drug use and pain presence suggests that medication effects alone are unlikely to fully explain the observed relationship, although the small sample size (*N* = 14) precludes firm conclusions. The underlying mechanisms, therefore, remain uncertain and warrant further investigation in longitudinal studies accounting for medication exposure. In addition, alexithymia emerged as an independent predictor of pain presence in our model. This finding is consistent with recent evidence in FMD populations highlighting the role of emotional processing difficulties in functional symptom expression [31] and is further supported by meta‐analytic data showing that alexithymia is elevated in chronic pain populations and relates to greater pain intensity, interference, and affective distress, suggesting a potential role of impaired emotional awareness in amplifying pain perception [32]. Finally, as expected, pain was also associated with poorer self‐reported physical health.

With respect to pain medication use, our data revealed that analgesic treatment was common among FMD patients. However, a closer examination of prescription and pain‐reporting patterns highlighted considerable heterogeneity: While a subset of patients reported no pain and were not taking analgesics, others reported pain despite receiving pharmacological treatment. Notably, a substantial proportion of patients experiencing pain were not taking any pain medication, but this should not automatically be interpreted as undertreatment, as the absence of analgesic therapy may also reflect contraindications, patient preference, or the use of non‐pharmacological management strategies (including psychological and physiotherapy interventions), which are often prioritized in chronic and functional pain conditions. Conversely, the finding that nearly 40% of patients reported pain despite taking analgesics suggests the potential inefficacy of the prescribed medications or a complex interplay between pain perception and FMD‐related mechanisms.

Some clinically relevant univariate findings did not remain significant in the multivariable model. For example, longer disease duration and combined or more severe motor presentations were associated with pain in univariate analyses but did not independently predict pain once other variables were considered simultaneously. These findings may still be clinically relevant, but suggest that their relationship with pain may be mediated by broader aspects of illness burden and perceived health status. Moreover, depressive symptoms were significantly higher in patients with pain at the group‐comparison level, but depression did not emerge as an independent predictor in the multivariable model: This may indicate that depressive symptoms overlap with other correlated dimensions, such as anxiety, fatigue, or poorer health‐related quality of life, rather than exerting an Independent contribution to pain presence. It is also possible that treatment differences, including the higher use of antidepressants in the pain group, may have influenced this pattern, although this possibility cannot be tested directly with the present data.

Finally, patients with FMD who did not report pain appeared to represent a clinically less complex subgroup, with fewer comorbidities—particularly lower rates of fibromyalgia—and better self‐reported physical health and overall outcomes. This is consistent with previous literature suggesting that the absence of pain is associated with a more favorable clinical profile. Therefore, this subgroup should not be interpreted as simply the opposite of the pain group, but rather represents a distinct and generally less burdensome clinical presentation.

### 4.2 Predictors of Pain Intensity and Interference in Patients With FMD

Among patients reporting pain at the BPI, our cohort exhibited a mean total score of 49.29 (maximum: 100), with average subscale scores of 18.63 for pain intensity (maximum: 40) and 30.66 for pain interference (maximum: 70). These values are in line with those reported in previous FMD cohorts using the same questionnaire, which documented pain intensity scores ranging from 18 to 21/40 and interference scores between 27 to 31/70 [33, 34].

In line with previous literature [6], our multivariable regression analyses showed that greater pain intensity and/or interference were associated with a range of clinical and dimensional factors, including RLS, IBS, anxiety symptoms, and poorer self‐rated physical and mental health.

Older patients reported slightly lower pain intensity, consistent with previous studies suggesting that thresholds for perceiving painful stimuli generally increase with age [35]. However, such results should be interpreted with caution, as: (1) statistical results were considerably below the MCID, representing the smallest change in a symptom score perceived as meaningful by the patient; (2) they might reflect changes in the dynamics of pain perception, modulation, and reporting in older individuals, rather than a genuine reduction in pain burden [36].

Restless Legs Syndrome was a significant predictor of higher pain intensity, with scores indicating a potentially clinically meaningful impact, overall suggesting that comorbid sensory–motor restlessness may amplify pain perception or reporting in FMD patients. The association between RLS and FMD has not been widely studied. Serranová et al. [37]. reported an increased prevalence of RLS in patients with FMD, associated with higher pain and sensory symptoms in lower limbs, when assessed with actigraphy, which might otherwise remain unrecognized in this population.

Similarly, IBS was associated with greater pain interference. This result is not unexpected, given that recurrent abdominal pain is part of the Rome IV diagnostic criteria for IBS [38] and in light of the well‐documented overlap between functional gastrointestinal disorders and heightened central pain sensitivity [39]. Neuroimaging studies in IBS [40] have demonstrated aberrant activation and functional connectivity in brain regions involved in interoception, affect, salience detection, and emotion regulation, correlating with both symptom severity and pain intensity. These findings suggest that in FMD, comorbid IBS may exacerbate the central amplification of pain or hypervigilance through shared neural circuitry, contributing to greater interference with daily functioning, although the clinical meaningfulness of this effect should be interpreted cautiously because the B±1 SE interval includes values below the prespecified MCID threshold.

The presence of physical trauma as a precipitating factor for FMD was linked to higher pain intensity. We did not assess the presence and intensity of pain at the time of the traumatic event, and it is uncertain whether pain itself played a causal role, acted as a reinforcing factor, or simply co‐occurred. Previous work proposed that physical events may contribute to the pathophysiology of FMD by enhancing attentional focus on bodily sensations and physical symptoms [2, 41]; our findings align with this hypothesis, although further research is needed to clarify the mechanisms linking physical trauma, pain, and functional symptoms.

Finally, among all predictors included in the model, self‐rated physical health had the largest independent contribution to both pain intensity and interference, followed by higher anxiety levels for pain intensity, consistent with literature showing that negative affect amplifies pain experience via attentional and emotional mechanisms [42] whereas lower self‐rated mental health independently predicted greater pain interference, indicating that broader psychological well‐being may be particularly relevant to the extent to which pain disrupts daily functioning.

### 4.3 Limits and Strengths

This study has several limitations. First, its cross‐sectional design prevents us from establishing the temporal relationship between pain and functional symptoms. Similarly, we cannot determine whether psychopathological symptoms were antecedents or consequences of the disorder.

Second, we did not collect data on the distribution or localization of pain across the body, which limits our ability to examine patterns of pain presentation in relation to specific motor phenotypes. Third, we lacked information on the timing and duration of pain symptoms, including whether the pain was acute, chronic, or fluctuating. This limitation is particularly relevant because the BPI captures pain during the 24 h prior to assessment; therefore, the prevalence estimates should be interpreted with caution. As a matter of fact, data were collected within a national registry assessing a broad range of comorbidities and psychopathological features, with pain representing one of several domains. As both acute (≤ 3 months) and chronic pain cases were included, the observed prevalence is likely higher and not directly comparable to studies focusing exclusively on chronic pain populations. Finally, our study focused exclusively on adult patients, limiting the generalizability of findings.

## 5 Conclusion

This study provides one of the most comprehensive characterizations to date of pain in patients with FMD, highlighting its high prevalence, clinical significance, and complex biopsychosocial correlates. We found that 78.8% of our patients reported experiencing pain as assessed over the previous 24 h, whose degrees of intensity and interference in daily life were associated with younger age, presence of RLS and IBS, increased anxiety, and lower self‐rated physical and mental health. Future longitudinal studies are needed to clarify causal relationships and to inform targeted pain management strategies.

## Author Contributions

**Francesco de Bertoldi:** conceptualization, investigation, writing – review and editing. **Lucia Tesolin:** conceptualization, investigation, writing – review and editing. **Roberto Eleopra:** conceptualization, investigation, writing – review and editing. **Benedetta Demartini:** conceptualization, methodology, formal analysis, writing – original draft, writing – review and editing, investigation. **Andrea Pilotto:** conceptualization, investigation, writing – review and editing. **Angela Sandri:** conceptualization, methodology, investigation, writing – review and editing. **Veronica Nisticò:** conceptualization, formal analysis, data curation, methodology, writing – original draft, writing – review and editing, investigation. **Luigi Michele Romito:** investigation, writing – review and editing, conceptualization. **Enrico Marcuzzo:** conceptualization, methodology, writing – review and editing, investigation. **Giovanni Mostile:** conceptualization, investigation, writing – review and editing. **Nicola Modugno:** conceptualization, investigation, writing – review and editing. **Enrica Olivola:** conceptualization, investigation, writing – review and editing. **Christian Geroin:** conceptualization, methodology, investigation, writing – original draft, writing – review and editing. **Alessandra Nicoletti:** conceptualization, investigation, writing – review and editing. **Tommaso Ercoli:** conceptualization, investigation, writing – review and editing. **Carlo Dallocchio:** conceptualization, investigation, writing – review and editing. **Roberto Erro:** conceptualization, investigation, writing – review and editing. **Alessandro Magliozzi:** conceptualization, investigation, writing – review and editing. **Roberto Ceravolo:** conceptualization, investigation, writing – review and editing. **Giovanni Defazio:** conceptualization, investigation, writing – review and editing. **Rosa De Micco:** conceptualization, investigation, writing – review and editing. **Marcello Esposito:** conceptualization, investigation, writing – review and editing. **Martina Petracca:** conceptualization, investigation, writing – review and editing. **Michele Tinazzi:** conceptualization, investigation, writing – original draft, writing – review and editing.

## Funding

The authors have nothing to report.

## Conflicts of Interest

The authors declare no conflicts of interest.

## Supporting information


**Appendix S1:** Co‐investigators of the Italian Registry of Functional Motor Disorders (IRFMDs) Study Group.

## Data Availability

The data that support the findings of this study are available from the corresponding author upon reasonable request.
