# Supplementary material for: Pain in Functional Motor Disorders: Clinical Correlates From the Italian Registry
Source: Eur J Neurol. 2026 May 18;33(5):e70634. doi: 10.1111/ene.70634 (PMC13182264; doi:10.1111/ene.70634)
Supplement: Supplementary file 1 — Appendix S1: Co‐investigators of the Italian Registry of Functional Motor Disorders (IRFMDs) Study Group. [file ENE-33-e70634-s001.docx]

**Supplementary Appendix S1. Co-investigators of the Italian Registry of Functional Motor Disorders (IRFMDs) Study Group**

| **Name** | **Location** | **Role** | **Contribution** |
| --- | --- | --- | --- |
| Paolo Amami | Neuropsychology Unit, Fondazione IRCCS Istituto Neurologico Carlo Besta, Milan, Italy. | Site Investigator | Site coordinator of data acquisition |
| Grazia Devigili | Fondazione IRCCS Istituto Neurologico Carlo Besta, Department of Clinical Neurosciences, Parkinson and Movement Disorders Unit, Milan, Italy. | Site Investigator | Site coordinator of data acquisition |
| Antonio E. Elia | Fondazione IRCCS Istituto Neurologico Carlo Besta, Department of Clinical Neurosciences, Parkinson and Movement Disorders Unit, Milan, Italy. | Site Investigator | Site coordinator of data acquisition |
| Nico-Golfrè Andreasi | Fondazione IRCCS Istituto Neurologico Carlo Besta, Department of Clinical Neurosciences, Parkinson and Movement Disorders Unit, Milan, Italy. | Site Investigator | Site coordinator of data acquisition |
| Francesco Teatini, | FND outpatients clinic, Neurology and Stroke Unit, General Hospital of Bolzano, Bolzano, Italy. | Site Investigator | Site coordinator of data acquisition |
| Chiara Sist | Neurology and Stroke Unit, General Hospital of Bolzano, Bolzano, Italy. | Site Investigator | Site coordinator of data acquisition |
| Igor Florio | Neurology and Stroke Unit, General Hospital of Bolzano, Bolzano, Italy. | Site Investigator | Site coordinator of data acquisition |
| Andreas Conca | Department of Psychiatry, General Hospital of Bolzano, Bolzano, Italy | Site Investigator | Site coordinator of data acquisition |
| Donatella Contrafatto | Department G.F. Ingrassia, Section of Neurosciences, University of Catania, Catania, Italy. | Site Investigator | Site coordinator of data acquisition |
| Eleonora Chisari | Department G.F. Ingrassia, Section of Neurosciences, University of Catania, Catania, Italy. | Site Investigator | Site coordinator of data acquisition |
| Francesca Conte | Department of Psychology, University of Milan, Milan, Italy | Site Investigator | Site coordinator of data acquisition |
| Giovanni Broglia | Aldo Ravelli Research Center for Neurotechnology and Experimental Brain Therapeutics, University of Milan, Milan, Italy. Department of Health Sciences, University of Milan, Milan, Italy. | Site Investigator | Site coordinator of data acquisition |
| Lorena Belli | IRCCS Neuromed, Pozzilli, Italy. | Site Investigator | Site coordinator of data acquisition |
| Alessandro Padovani | Department of Clinical and Experimental Sciences, Neurology Unit, University of Brescia, Italy. Laboratory of Digital Neurology and Biosensors, University of Brescia, Italy. Neurology Unit, Department of Continuity of Care and Frailty, ASST Spedali Civili Brescia Hospital, Italy. Neurobiorepository and Laboratory of Advanced Biological Markers, University of Brescia and ASST Spedali Civili Hospital, Brescia, Italy. Brain Health Center, University of Brescia, Italy. | Site Investigator | Site coordinator of data acquisition |
| Cinzia Zatti | Department of Clinical and Experimental Sciences, Neurology Unit, University of Brescia, Italy. Laboratory of Digital Neurology and Biosensors, University of Brescia, Italy. Neurology Unit, Department of Continuity of Care and Frailty, ASST Spedali Civili Brescia Hospital, Italy. Neurobiorepository and Laboratory of Advanced Biological Markers, University of Brescia and ASST Spedali Civili Hospital, Brescia, Italy. | Site Investigator | Site coordinator of data acquisition |
| Carla Piano | Fondazione Policlinico Universitario Agostino Gemelli IRCCS, Rome, Italy.  Department of Neuroscience, Università Cattolica del Sacro Cuore, Rome, Italy. | Site Investigator | Site coordinator of data acquisition |
| Paola Zinzi | Fondazione Policlinico Universitario Agostino Gemelli IRCCS, Rome, Italy. | Site Investigator | Site coordinator of data acquisition |
| Carla Arbasino | SC Neurologia, Dipartimento di Area Medica Specialistica, ASST Pavia, Pavia, Italy. | Site Investigator | Site coordinator of data acquisition |
| Giovanna Savorgnan | SC Neurologia, Dipartimento di Area Medica Specialistica, ASST Pavia, Pavia, Italy. | Site Investigator | Site coordinator of data acquisition |
| Ferdinando Ambrosio | Department of Advanced Medical and Surgical Sciences, University of Campania “Luigi Vanvitelli”, Naples, Italy. | Site Investigator | Site coordinator of data acquisition |
| Oriana Ciaramaglia | Department of Advanced Medical and Surgical Sciences, University of Campania “Luigi Vanvitelli”, Naples, Italy. | Site Investigator | Site coordinator of data acquisition |
| Trinchillo Assunta | Department of Medical, Motor and Wellness Sciences, University "Parthenope", Naples, Italy | Site Investigator | Site coordinator of data acquisition |
| Habteswallner Francesco | Clinical Neurophysiology Unit, Cardarelli Hospital, Naples, Italy | Site Investigator | Site coordinator of data acquisition |
| Sofia Cuoco | Center for Neurodegenerative Diseases (CEMAND), Department of Medicine, Surgery and Dentistry Scuola Medica Salernitana, University of Salerno, Baronissi (SA), Italy. | Site Investigator | Site coordinator of data acquisition |
| Immacolata Carotenuto | Center for Neurodegenerative Diseases (CEMAND), Department of Medicine, Surgery and Dentistry Scuola Medica Salernitana, University of Salerno, Baronissi (SA), Italy. | Site Investigator | Site coordinator of data acquisition |
| Eleonora Del Prete | Centro Clinico Malattie NeuroDegenerative-Azienda Ospedaliero Universitaria Pisana | Site Investigator | Site coordinator of data acquisition |
| Enrico Bergamin | Centro Clinico Malattie NeuroDegenerative-Azienda Ospedaliero Universitaria Pisana | Site Investigator | Site coordinator of data acquisition |
| Maurizio Zibetti | Department of Neurosciences Rita Levi Montalcini, University of Turin, Turin, Italy  SC Neurologia 2 U, AOU Città della Salute e della Scienza, Torino, Italy. | Site Investigator | Site coordinator of data acquisition |
| Anny Votano | Center for Botulinum Toxin Therapy, University Hospital, Catanzaro, Italy | Site Investigator | Site coordinator of data acquisition |
| Giovanni Fabbrini | IRCCS Neuromed, Pozzilli, Italy; Department Human Neurosciences, Sapienza University of Rome, Rome, Italy | Site Investigator | Site coordinator of data acquisition |
